# Whole Exome Sequencing Reveals Homozygous Mutations in *RAI1*, *OTOF*, and *SLC26A4* Genes Associated with Nonsyndromic Hearing Loss in Altaian Families (South Siberia)

**DOI:** 10.1371/journal.pone.0153841

**Published:** 2016-04-15

**Authors:** Alexander Y. Сhurbanov, Tatiana M. Karafet, Igor V. Morozov, Valeriia Yu. Mikhalskaia, Marina V. Zytsar, Alexander A. Bondar, Olga L. Posukh

**Affiliations:** 1 Arizona Research Laboratories, Division of Biotechnology, University of Arizona, Tucson, Arizona, United States of America; 2 SB RAS Genomics Core Facility, Institute of Chemical Biology and Fundamental Medicine, Siberian Branch of the Russian Academy of Sciences, Novosibirsk, Russian Federation; 3 Novosibirsk State University, Novosibirsk, Russian Federation; 4 Laboratory of Human Molecular Genetics, Federal Research Center Institute of Cytology and Genetics, Siberian Branch of the Russian Academy of Sciences, Novosibirsk, Russian Federation; CNR, ITALY

## Abstract

Hearing loss (HL) is one of the most common sensorineural disorders and several dozen genes contribute to its pathogenesis. Establishing a genetic diagnosis of HL is of great importance for clinical evaluation of deaf patients and for estimating recurrence risks for their families. Efforts to identify genes responsible for HL have been challenged by high genetic heterogeneity and different ethnic-specific prevalence of inherited deafness. Here we present the utility of whole exome sequencing (WES) for identifying candidate causal variants for previously unexplained nonsyndromic HL of seven patients from four unrelated Altaian families (the Altai Republic, South Siberia). The WES analysis revealed homozygous missense mutations in three genes associated with HL. Mutation c.2168A>G (*SLC26A4*) was found in one family, a novel mutation c.1111G>C (*OTOF*) was revealed in another family, and mutation c.5254G>A (*RAI1*) was found in two families. Sanger sequencing was applied for screening of identified variants in an ethnically diverse cohort of other patients with HL (n = 116) and in Altaian controls (n = 120). Identified variants were found only in patients of Altaian ethnicity (n = 93). Several lines of evidences support the association of homozygosity for discovered variants c.5254G>A (*RAI1*), c.1111C>G (*OTOF*), and c.2168A>G (*SLC26A4*) with HL in Altaian patients. Local prevalence of identified variants implies possible founder effect in significant number of HL cases in indigenous population of the Altai region. Notably, this is the first reported instance of patients with *RAI1* missense mutation whose HL is not accompanied by specific traits typical for Smith-Magenis syndrome. Presumed association of *RAI1* gene variant c.5254G>A with isolated HL needs to be proved by further experimental studies.

## Introduction

Hearing loss (HL) is one of the most common sensorineural disorders, affecting one in 500–1000 newborns. Approximately half of these cases have an underlying genetic basis for their HL [1]. More than 400 genetic syndromes have been associated with HL, and almost 80% of familial HL is nonsyndromic hearing loss (NSHL). Hereditary HL can be inherited in an autosomal dominant, autosomal recessive, or X-linked recessive manner as well as by mitochondrial inheritance [2]. Mutations in gene *GJB2* (gap junction protein, beta-2, MIM 121011) encoding connexin 26 (Cx26) account for a significant portion (up to 50%) of autosomal recessive NSHL (ARNSHL) among various ethnic groups [1,2].

Despite significant progress in identifying deafness-related genes, the genetic causes of inherited NSHL often remain unclear due to extreme ethnicity-specific variation and limited phenotypic variability. Current genetic testing applies Sanger sequencing and microarray approaches primarily for detecting mutations in *GJB2* and for a small number of other known common deafness genes. The genetic heterogeneity of HL—with ~ 140 genetic loci currently associated with NSHL and many others involved in HL [3]—makes these approaches impractical for genetic testing of individual patients. More recently, the rapid development of next-generation sequencing (NGS) methods including whole-exome sequencing (WES) have enabled researchers to identify unknown deleterious variants in a significant number of HL cases [4–9]. One of the strategies for searching new deafness-associated candidate genes and variants combines preliminary linkage analysis with subsequent NGS for narrowed chromosomal regions [6–8]. However, linkage analyses are hardly applicable for affected patients from small-size non-consanguineous families. In such cases application of WES allows interrogation of all protein-coding genome regions in a single experiment [9].

In previous work we investigated the molecular basis of deafness by screening *GJB2* (Cx26) mutations in ethnically heterogeneous patients from the Altai Republic (South Siberia) and found variable contribution of certain mutations in *GJB2* to HL in patients belonging to different ethnic groups [10]. Potential genetic causes for deafness in patients with the absence of *GJB2* deleterious mutations remained unclear. The aim of the present work is to apply WES to investigate contributing genetic factors for hearing impairment in HL patients living in the Altai Republic.

## Materials and Methods

### Patients and controls

A cohort of 163 ethnically heterogeneous patients with HL from the Altai Republic was collected during field work between 2002 and 2011 and included 93 indigenous Altaians, 33 Russians, 13 Kazakhs, and 24 individuals of mixed and other ethnicities. Preliminary screening of *GJB2* (Cx26) mutations revealed deleterious *GJB2* mutations in 40 out of 163 examined patients and their contribution to HL varied from 15.1% in Altaian patients to 51.5% in Russians (OLP, unpublished data). Causes for deafness in Cx26-negative patients (n = 123, including 79 Altaians, 16 Russians, 10 Kazakhs, and 18 individuals of mixed and other ethnicities) remained unknown.

We explored the utility of WES for identifying candidate causal variants in seven Cx26-negative for deleterious variants Altaian patients with congenital profound HL of unknown genetic etiology from four unrelated extended families (F38, F40, F53, and F54) (see S1 Table). Multiple affected siblings in each family along with unaffected parents allowed us to infer an autosomal recessive mode of HL inheritance in these families. These families live in remote small villages in various administrative districts of the Altai Republic. Hearing status of affected individuals was evaluated by otoscopic and pure-tone audiometry examinations, which patients underwent at different times in the only specialized audiologic service in the Altai Republic. Other concomitant information was collected from local unspecialized medical services and by direct interview with the patients and their relatives.

120 normal hearing unrelated Altaians were also included in this study as controls.

### Ethics Statement

Written informed consent was obtained from all individuals who participated in this study or their legal guardians. This study was approved by the Ethics Committee at the Institute of Cytology and Genetics (Novosibirsk, Russian Federation) and was in compliance with the Declaration of Helsinki.

### Whole exome sequencing (WES) and sequence data analysis

Genomic DNA was isolated by a standard phenol chloroform extraction method. Seven genomic DNA samples were sequenced on Illumina HiSeq 2000 using Agilent SureSelect Human All Exon V4 51Mb enrichment kit. Sequence reads generated from the libraries were filtered for quality, aligned and mapped to the hg19 human reference genome using the gsNap program [11]. The variant calling process for both indels (insertion/deletions) and single nucleotide variants was done by using the Genome Analysis Toolkit (GATK, http://www.broadinstitute.org/gatk). ANNOVAR software was applied for variant functional annotation [12]. The deleterious impact of non-synonymous SNPs was predicted by the PolyPhen-2 (version 2.2.2) (http://genetics.bwh.harvard.edu/pph2) [13]. Under the assumption that HL in siblings from families F38, F40, and F54 was likely caused by the same mutations in corresponding gene with recessive pattern of inheritance, we focused our evaluation of the WES data sets only on homozygous or compound heterozygous variants shared by affected siblings from the same family. The only patient from family F53 was screened for rare deleterious variants known as related with HL according to ClinVar (http://www.ncbi.nlm.nih.gov/clinvar/). Additional criteria for filtering variants was an allele frequency < 5%. All shared variants predicted by PolyPhen-2 as “possibly damaging” or “probably damaging” were further analyzed for zygosity in the context of a candidate gene list for NSHL genes with known autosomal recessive inheritance patterns [3]. To extend the candidate gene list we have also searched OMIM records (http://www.omim.org/) for the terms “hearing loss” and “deafness”. Rarity of the variants was predicted based on the alternate allele frequency according to the dbSNP138 (http://www.ncbi.nlm.nih.gov/projects/SNP/), 1000 Genomes Project (http://www.1000genomes.org/), Exome Sequencing Project (ESP, 6500 exomes, http://evs.gs.washington.edu/EVS/), and Exome Aggregation Consortium (ExAC, Cambridge, MA, http://exac.broadinstitute.org).

### Sanger sequencing

Three mutations detected by WES in seven Altaian patients from four families F38, F40, F53, and F54 were validated by Sanger sequencing. Cohort of all other Cx26-negative patients (n = 116, including all available members from the WES families), and 120 normal hearing Altaians were further screened by Sanger sequencing for the identified variants in the *RAI1*, *OTOF*, and *SLC26A4* genes. Primer pairs designed to amplify corresponding PCR products and used also as Sanger primers are presented in S2 Table. PCR products were purified using Agencourt Ampure XP.

The coding region of the *RAI1* gene encompassing exons 3, 4, 5 and part of exon 6 (mRNA NCBI Reference Sequence: NM_030665.3) with flanking intronic regions was sequenced in 13 individuals heterozygous or homozygous for mutation c.5254G>A (*RAI1*). Primers and PCR conditions are available upon request.

Sanger sequencing was performed using an Applied Biosystems BigDye Terminator V.3.1 Cycle Sequencing Kit, with subsequent unincorporated dyes removal by Sephadex G-50 gel filtration. Sanger products were analyzed on an Applied Biosystems 3130xl Genetic analyzer.

### Statistical methods

Two-tailed Fisher’s exact test with significance level of p<0.05 was applied to compare allele frequencies between patients and controls.

## Results

The results of WES, variants filtering and evolutionary conservation for the positions of detected variants are presented in Table 1 and S1 Fig. A novel homozygous missense mutation c.5254G>A (p.Gly1752Arg) in gene *RAI1* (retinoic acid induced 1, MIM 607642) was found in two pairs of WES siblings from families F38 and F40; a novel homozygous missense mutation c.1111C>G (p.Gly371Arg) in gene *OTOF* (otoferlin, MIM 603681) was observed in both WES siblings from family F54; and a previously known homozygous missense mutation c.2168A>G (p.His723Arg) was found in gene *SLC26A4* (pendrin, MIM 605646) in the only WES examined affected individual from family F53 (Table 2). It is interesting to note that the *RAI1* gene is absent in the candidate gene list for genes associated with nonsyndromic and syndromic HL whereas *OTOF* and *SLC26A4* are known as deafness-related genes [3]. The presence of three detected mutations was further tested by Sanger sequencing in all available members from families F38, F40, F53, and F54. These candidate variants were also screened in all other Cx26-negative patients with HL and in the Altaian control group.

**Table 1:** The results of the WES analysis and variants filtering for seven patients.

	Family ID and patient code						
	F38		F40		F53	F54	
	38-II-4	38-II-5	40-II-1	40-II-3	53-II-1 ^a^	54-II-2	54-II-5
Average depth of coverage (X)	41.35	40.55	49.10	35.58	47.62	28.27	45.93
Number of variants in CDS	50,204	49,728	52,813	45,825	53,823	44,995	53,851
Rare non-synonymous shared variants	1,235		1,428		2,578	1,437	
Rare non-synonymous shared homozygous variants	139		151		332	173	
Rare non-synonymous shared homozygous variants in candidate genes	1		1		6	1	
Rare non-synonymous shared homozygous variants in candidate genes predicted deleterious	1		1		1	1	

^a^—The only one patient from family F53 (53-II-1) was analyzed by WES. There are no rare shared indels.

**Table 2:** Missense variants identified in individuals studied by WES.

	Patient code		
	38-II-4, 38-II-5, 40-II-1, 40-II-3	54-II-2, 54-II-5	53-II-1
Chromosome and reference position (in hg19)	chr17:17701516(G)	chr2:26707436(C)	chr7:107350577(A)
Gene (exon)	*RAI1* (exon 3)	*OTOF* (exon 12)	*SLC26A4* (exon 19)
Nucleotide change (Amino acid change)	c.5254G>A (p.Gly1752Arg) *	c.1111C>G (p.Gly371Arg) *	c.2168A>G (p.His723Arg)
Accession number	NM_030665.3	NM_194248.2	NM_000441.1
dbSNP138 (Global MAF)	rs755572135 (no info) **	-	rs121908362 (G = 0.0004/1)
1000 Genome Project database, alt. freq.	-	-	0.0009
Exome Sequencing Project (ESP) 6500 exomes	-	-	-
Exome Aggregation Consortium (ExAC): allele number (allele freq.)	11: 107,784 (0.0001021)	-	15: 121,166 (0.0001238)
PolyPhen2 HumVar score	0.674 (possibly damaging)	1.0 (probably damaging)	1.0 (probably damaging)
SIFT	tolerated	damaging (0.0)	deleterious (0.0)
Mutation Taster	polymorphism	disease_causing (0.0)	disease_causing (0.0)
LRT	deleterious (0.000124)	deleterious (0)	deleterious (0)
PhyloP Score (100 vertebrates)	1.308	7.701	6.299

* These sequence variants were submitted to ClinVar (http://www.ncbi.nlm.nih.gov/clinvar/) and the ClinVar accession numbers for the NM_030665.3: Chr.17_17701516_17701516_G_A and the NM_194248.2: Chr.2_26707436_26707436_C_G sequences are SCV000196150 and SCV000196151, respectively.

**—submitted by Genetic Services Laboratory, University of Chicago (Sept. 15, 2015).

### Variant c.5254G>A (p.Gly1752Arg) in the *RAI1* gene

**Fig 1:**
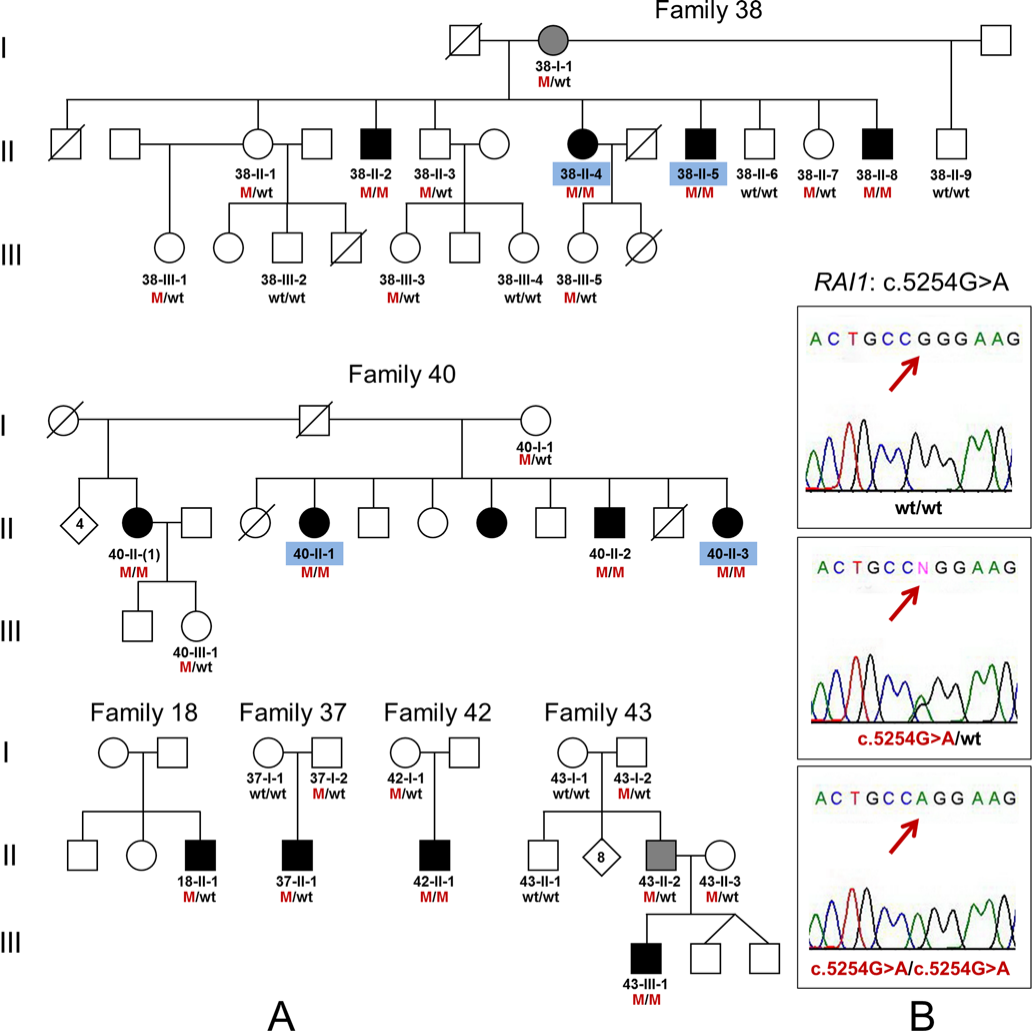
Identification of the c.5254G>A (p.Gly1752Arg) mutation in the *RAI1* gene in Altaian families. (**A**) Pedigrees of the WES families F38, F40, and additional four Altaian families F18, F37, F42, and F43 with mutation c.5254G>A (p.Gly1752Arg) in the *RAI1* gene. (**B**) Validation of c.5254G>A by Sanger sequencing. WES was performed for affected subjects indicated by blue codes. Black symbols represent individuals with congenital profound HL, moderate or severe HL in individuals is marked by grey symbols. M—mutation c.5254G>A (p.Gly1752Arg), wt—wild type.

Cosegregation of homozygosity for mutation c.5254G>A in the *RAI1* gene with congenital bilateral profound HL was confirmed in the WES families F38 and F40 (Fig 1). The *RAI1* gene is known as the primary gene for Smith-Magenis syndrome (SMS, MIM 182290) (prevalence 1:25,000), which is characterized by variable intellectual disability including speech and motor delay, behavioral abnormalities like self-injurious and/or aggressive behavior, sleep disturbance, particular craniofacial and skeletal abnormalities, obesity, hearing loss, hoarse voice and other characteristic traits. About 90% of SMS cases are known to be associated with different deletions (ranging from 1.5 to 9 Mb, with the most common ∼ 3.7 Mb) of chromosome 17p11.2 region, which contains several genes including the *RAI1* gene [14–16]. Approximately 10% of patients with SMS clinical features are associated with heterozygous *RAI1* mutations [17–22].

Affected members from families F38 and F40 with c.5254G>A (*RAI1*) were originally examined in 2002. After performing WES we revisited these families in 2013 to verify the presence of a nonsyndromic type of HL in affected family members. We found that all affected members of both families F38 and F40 did not have the additional symptoms typical for SMS patients with mutations in *RAI1*. In particular, we did not observe any craniofacial and skeletal abnormalities (brachycephaly, midface hypoplasia, broad square-shaped face, a tented upper lip, deep-set eyes, brachydactylia, short stature) and obesity. Close relatives denied presence or history of the behavioral abnormalities (self-injurious and/or aggressive behavior) and sleep disturbances in the patients with mutation c.5254G>A. All patients have not shown evident intellectual disability. They all attended at different time the special school for deaf and hard of hearing children and there were no other indications for their learning difficulties strongly distinguishing them from other deaf students. All patients use only sign language for communication and we were unable to recognize whether they have a hoarse-deep voice typical for SMS or not. Mother in family F38 (38-I-1) heterozygous for c.5254G>A (Fig 1) had no hearing complaints during first examination in 2002 when she was 65 years old. Reevaluation of her clinical status eleven years later, however, revealed moderate-to-severe HL with obvious difficulties in verbal communication. Due to a strong personal belief that her hearing impairment emerged in recent years due to hypertension, she declined additional audiological examination.

Mutation c.5254G>A was also found in additional five patients from four unrelated Altaian families F18, F37, F42, and F43 (Fig 1). Subjects homozygous for c.5254G>A (42-II-1 and 43-III-1) had congenital profound sensorineural HL. Subjects heterozygous for c.5254G>A (18-II-1, 37-II-1, and 43-II-2) had varying onset and degree of HL: profound HL developed at the age of 8 months after an unspecified illness was observed in patient 18-II-1; severe HL was observed in patient 37-II-1 and was apparently influenced by perinatal asphyxia; unilateral moderate HL was observed in patient 43-II-2 during adulthood and was partly attributed to chronic otitis media. None of additional five patients with c.5254G>A demonstrated any signs or symptoms consistent with SMS. In a control group of 120 unrelated, normal-hearing Altaians four individuals were heterozygous c.5254G>A carriers (4/120, 3.33%).

To search potential additional deleterious variants in compound with c.5254G>A in affected heterozygotes for c.5254G>A and to evaluate the *RAI1* allelic variations with c.5254G>A, we sequenced the *RAI1* gene coding region encompassing exons 3, 4, 5 and part of exon 6 with flanking intronic regions in 13 individuals homozygous or heterozygous for c.5254G>A. These individuals included four affected homozygotes (38-II-5, 40-II-1, 42-II-1, 43-III-1), four affected heterozygotes (18-II-1, 37-II-1, 38-I-1, 43-II-2), five normal hearing heterozygotes including three relatives (37-I-2, 43-II-3, 38-II-1) (see Fig 1), and two carriers of c.5254G>A from Altaian controls whose DNAs were available (Alt-1, Alt-2). The sequencing results are presented in S3 Table. No additional deleterious *RAI1* variants were found in compound with c.5254G>A in affected heterozygotes for c.5254G>A (18-II-1, 37-II-1, 38-I-1, and 43-II-2). Variations in sequenced region were defined by known SNPs (rs3803763, rs11649804, rs8067439) and variable poly(CAG) region (including rs110783980) starting from 832 nucleotide position (S3 Table). The only combination of two identical alleles_mut_: C-A-Q13[CAG CAA (CAG)10 del(CAG) CAA]-G-**c.5254G>A** was observed in all c.5254G>A homozygote which was suggested as the common allelic haplotype for c.5254G>A. For all c.5254G>A heterozygotes, the haplotypes were reconstructed by singling out allele_mut_ with assignment of residuary nucleotide variants to the second allele. For members of families F37 and F43 (37-II-1 and 37-I-2, 43-II-2 and 43-II-3, respectively) the haplotypes were confirmed by corresponding pedigree data. In total, five different *RAI1* alleles were reconstructed (S3 Table). All studied individuals with mutation c.5254G>A share a specific allele_mut_: C-A-Q13[CAG CAA (CAG)10 del(CAG) CAA]-G-**c.5254G>A** suggesting the common origin of c.5254G>A in Altaians.

Mutation c.5254G>A was found only in Cx26-negative patients of Altaian ethnicity. For accurate estimation of c.5254G>A allelic frequency, we also screened this mutation in Cx26-positive Altaian patients with HL. Nobody from this group had c.5254G>A. We compared frequency of c.5254G>A in a total sample of Altaian patients (0.129, 24/186 chromosomes) and in a control group of normal hearing Altaians (0.017, 4/240 chromosomes). We found significantly (p<10^−5^) higher frequency of c.5254G>A in Altaian patients. To avoid probable bias due to known presence of related individuals in the total cohort of Altaian patients, we selected only unrelated patients (n = 74) by pedigree analysis and compared the c.5254G>A frequency in this group (0.081, 12/148 chromosomes) with the frequency in the control sample. Again, a statistically higher c.5254G>A frequency (p = 0.0026) was observed among selected patients. This observation supports a presumed association of mutation c.5254G>A (*RAI1*) with HL.

### Variant c.1111C>G (p.Gly371Arg) in the *OTOF* gene

**Fig 2:**
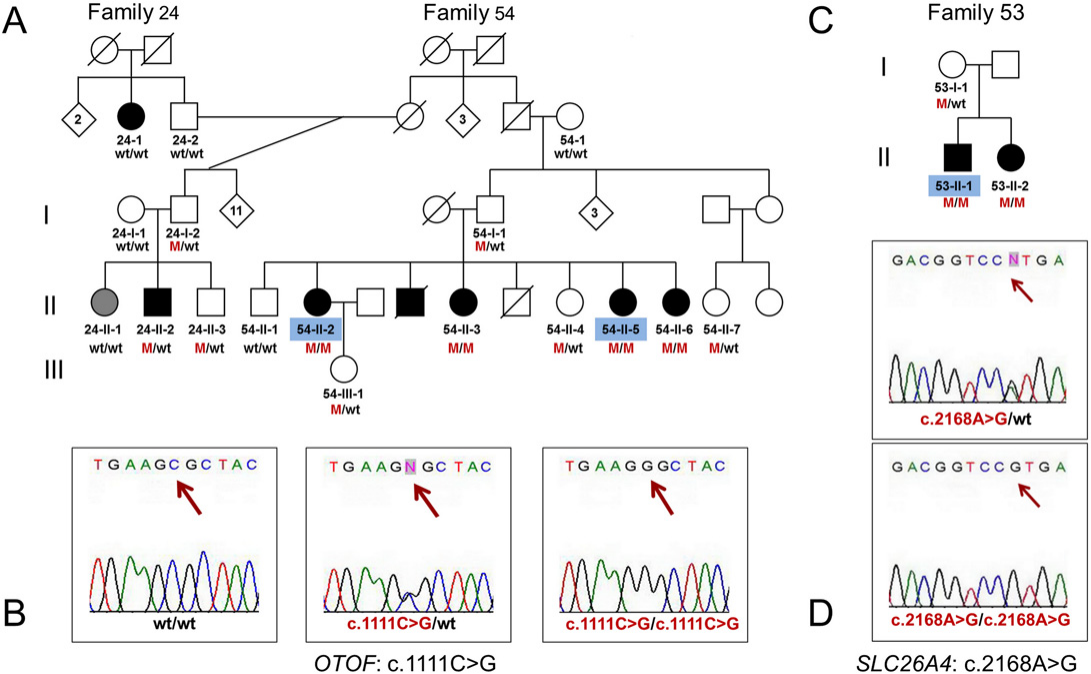
Identification of the c.1111C>G (p.Gly371Arg) mutation in the *OTOF* gene and the c.2168A>G (p.His723Arg) mutation in the *SLC26A4* gene in Altaian families. (**A**) Extended pedigree of the WES family F54 (together with related family F24) with mutation c.1111C>G (p.Gly371Arg) in the *OTOF* gene denoted as M. (**B**) Validation of c.1111C>G by Sanger sequencing. (**C**) Pedigree of the WES family F53 with mutation c.2168A>G (p.His723Arg) in the *SLC26A4* gene which is denoted as M. (**D**) Validation of c.2168A>G by Sanger sequencing. WES was performed for affected subjects indicated by blue codes. Black symbols represent individuals with congenital profound HL; grey symbol represents individual 24-II-1 with moderate or severe HL. wt—wild type.

We found a novel mutation c.1111C>G (p.Gly371Arg) in the *OTOF* gene in two siblings (54-II-2, 54-II-5) from Altaian family F54 with congenital bilateral profound sensorineural HL (Fig 2). The *OTOF*, encoding the transmembrane protein otoferlin, is one of the NSHL-related genes associated with autosomal recessive inheritance patterns of HL [3]. Segregation of homozygosity for c.1111C>G with HL was confirmed by Sanger sequencing in other affected siblings (54-II-3, 54-II-6). All tested normal hearing relatives were either wt/wt (54–1 and 54-II-1) or heterozygous for c.1111C>G (54-I-1, 54-II-4, 54-II-7, and 54-III-1).

Sanger sequencing in other Cx26-negative patients revealed heterozygosity for c.1111C>G in individual 24-II-2 with prelingual profound sensorineural HL from Altaian family F24, which was closely related to F54 (Fig 2). Analysis of the combined pedigree F24-F54 led us to conclude that the cause of HL in patient 24-II-2 was unlikely to be associated with the presence of a single copy of c.1111C>G: the only individual 24-II-2 out of seven detected c.1111C>G heterozygotes (24-I-2, 24-II-2, 24-II-1, 54-I-1, 54-II-4, 54-II-7, 54-III-1,) was affected and c.1111C>G was absent in his relatives 24–1 (with congenital profound HL) and 24-II-1 (with moderate HL). On the other hand, all homozygotes for c.1111C>G (54-II-2, 54-II-3, 54-II-5, 54-II-6) were affected. We assume that other factors (genetic or environmental) could be involved in HL development in individuals belonging to branch F24 of combined pedigree F24-F54. Mutation c.1111C>G was not found among 120 normal hearing Altaians.

### Variant c.2168A>G (p.His723Arg) in gene *SLC26A4*

Homozygous mutation c.2168A>G (p.His723Arg) in gene *SLC26A4* was found in the only patient 53-II-1 from Altaian family F53 who was tested by WES (S1 Table). Subsequent Sanger sequencing confirmed homozygous c.2168A>G in his affected sister (53-II-2) and heterozygous c.2168A>G state in their normal hearing mother (53-I-1) (Fig 2).

The *SLC26A4* gene encodes pendrin, an iodide/chloride/bicarbonate transporter, expressed in the inner ear, thyroid, kidney, salivary duct, and respiratory tract. Recessive mutations in *SLC26A4* are associated with Pendred syndrome (PDS, MIM 274600) which combines HL and goiter, and autosomal recessive deafness with enlarged vestibular aqueduct (EVA) (DFNB4, MIM 600791) and/or incomplete partition of the cochlea (i.e. Mondini dysplasia) [23,24]. Both homozygous for c.2168A>G siblings (53-II-1 and 53-II-2, born in 1988 and 1989, correspondingly) were affected with congenital bilateral profound sensorineural HL diagnosed by routine otoscopic and audiological examination in 1994. Presence or absence of EVA in these patients was not confirmed due to the unavailability of temporal bone computed tomography examination. Mutation c.2168A>G was found neither in other Cx26-negative patients nor among 120 normal hearing Altaians.

## Discussion

We applied WES for identification of candidate causal variants in some Altaian families with autosomal recessive NSHL of unknown genetic etiology and identified homozygous missense mutations in three genes (*RAI1*, *OTOF*, and *SLC26A4*) associated with HL.

Our study presents the first reported case of the patients with missense mutation in the *RAI1* gene whose HL is not accompanied by specific traits (variable intellectual disability, speech and motor delay, self-injurious and/or aggressive behavior, sleep disturbance, particular craniofacial and skeletal abnormalities, obesity, hearing loss, hoarse voice and other less common characteristic traits) typical for Smith-Magenis syndrome (SMS). The SMS cases are known to be associated with different deletions in chromosome 17p11.2 region, which contains several genes including the *RAI1* gene (~ 90% of cases) [14–16] and with heterozygous *RAI1* mutations (~ 10% of cases) [17–22]. Haploinsufficiency of *RAI1* is suggested to be responsible for most SMS features, while other genes located in the SMS region are probably associated with varying severity of the SMS phenotype. Different SMS mouse models have recapitulated some common traits of SMS phenotypes including a range of variable penetrance of craniofacial defects, obesity, behavioral abnormalities, circadian abnormalities [25–27]. Additionally, the *RAI1* gene is associated not only with Smith-Magenis syndrome but also with some other neurodegenerative and neuropsychiatric disorders [28–33]. Up till now, the involvement of *RAI1* in auditory pathways is unknown.

The exact functional role of the RAI1 protein is not known though several lines of evidence indicate that RAI1 is a potential transcription factor and may be involved in development cell growth and cell cycle regulation, neurobehavioural and circadian rhythm pathways [17,18,34–37]. Human *RAI1* gene has been shown to be very similar to its mouse ortholog both in DNA and protein sequences and in expression patterns [38,39]. Murine *Rai1* is mainly expressed in brain tissue though its expression was also detected in multiple organs and tissues with variable expression levels in different subtypes of tissues and cells [38]. Recently, Fragoso *et al* [40] demonstrated specific distribution of RAI1 protein in multiple regions of the human brain involved in cognitive and motor functions. In these regions, RAI1 was differentially expressed in neurons and its subcellular distribution implied both nuclear and cytoplasmic localization [40].

About 60–68% of all SMS patients (with deletions of chromosome 17p11.2 region or with mutations in *RAI1*) have at least some degree of hearing impairment, which can be conductive, sensorineural, or mixed in nature. However, the etiology of HL (environmental or genetic factors) in the SMS patients is not yet clearly established [14–16,34,41]. One exception is the case presented by Liburd *et al* [42] where moderately severe high-frequency hearing loss in one of eight SMS patients with common deletion was actually caused by missense mutation in the *MYO15A* gene which is responsible for nonsyndromic autosomal recessive profound hearing loss DFNB3 [3]. Gene *MYO15A* locates in the SMS region at 17p11.2 and thereby this patient was hemizygous for *MYO15A* [42].

Our current data suggest a possible association of homozygous *RAI1* missence mutation detected in this study, c.5254G>A, with isolated HL in Altaian patients. This mutation was previously found with extremely low frequency (11: 107 784 alleles, ExAC data) (Table 2). Conservative in many organisms (S1 and S2 Figs), this variant leads to changing nonpolar glycine to basic polar arginine (p.Gly1752Arg) at amino acid position 1752 in the C-terminal part of RAI1. Variant c.5254G>A has been predicted ‘possibly damaging’ by PolyPhen2 [13] and ‘deleterious’ by LRT [43], but other programs like SIFT [44] and Mutation Taster [45] predicted it as benign polymorphism (Table 2). Among these tools Polyphen2 prediction has a priority as it considers violations in protein structure associated with the variant and is currently used in most of the NGS annotations pipelines. All detected c.5254G>A homozygotes (in total, 10 patients from 6 unrelated families) were affected by nonsyndromic HL (Fig 1). At least for 4 patients (from families F38 and F40) no other known HL-associated mutations were found by whole exome sequencing. The possibility of another allelic mutation in *RAI1* gene in four affected c.5254G>A heterozygous individuals (from total 20 detected heterozygotes) was excluded by sequencing the *RAI1* coding region and we speculate that HL in these patients could be caused by the other reasons (most likely environmental factors according to their medical histories). The association implies significant variant enrichment in disease cases compared to matched control and we indeed detected significantly higher frequency of c.5254G>A in Altaian patients compared with Altaian controls. In addition, high prevalence of variant c.5254G>A in the Altaians is probably due to founder effect in small isolated Altaian population: the presence of only *RAI1* allele_mut_: C-A-Q_13_[CAG CAA (CAG)_10_ del(CAG) CAA]-G-**c.5254G>A** was shared by all studied carriers of c.5254G>A that supports the common origin of c.5254G>A in Altaians.

Heterozygous *RAI1* mutations were found in approximately 10% of the SMS patients [17–22]. All known mutations in *RAI1* detected in SMS patients with relevant references are summarized in S4 Table and S2 Fig. The majority of reported *RAI1* mutations are located in exon 3, which contains ~ 98% of the *RAI1* gene coding sequence [39]. Hearing impairment along with other traits of SMS is observed at least in 25% of SMS patients with *RAI1* mutations (see details in S4 Table). Most of the mutations detected in SMS patients are *de novo* dominant nonsense or frameshift mutations resulting in truncated protein RAI1. Functional analysis of several truncated forms of the RAI1 protein revealed that the N-terminal half of the protein (1–1034 aa) has transactivational activity, while the C-terminal half is responsible for its transportation into the nucleus, and both are essential for proper function of RAI1 [43,44]. No significant differences were found in the clinical phenotype of SMS patients carrying nonsense or frameshift *RAI1* mutations either in the N-terminal or the C-terminal half of the RAI1 protein [43]. To date, only several missense mutations in *RAI1* are known (S4 Table) and their deleterious mechanism is not yet clearly established. The mutated forms of RAI1 protein with missense mutations p.R1217Q (c.3650G>A), p.Q1389R (c.4166A>G), p.Q1562R (c.4685A>G) and p.S1808N (c.5423G>A) (all are in C-terminal half of the RAI1 protein) were originally reported to have the same transactivation activity and nucleus location as wild type RAI1 [22,46]. In a more recent investigation, Carmona-Mora *et al* [47] discovered that these missense mutations lead to diminishing activation driven by the BDNF enhancer when compared to the wild type RAI1 protein. Two possible explanations were suggested: (i) the presence of the mutant amino acids leads to impairment of direct (or indirect) DNA binding site within the C-terminal region or (ii) the regulatory domain that is present in this part of the protein is negatively affecting the transcription factor activity [47]. Mutation found in our study, p.Gly1752Arg, locates in RAI1 amino acid sequence between mutations p.Q1562R (c.4685A>G) and p.S1808N (c.5423G>A) studied by Carmona-Mora *et al* [47] therefore we speculate that two aforesaid possible explanations [47] might also be applicable for p.Gly1752Arg.

Our findings meet main genetic criteria [48] for classifying p.Gly1752Arg variant as pathogenic rather than benign: (i) strong segregation of homozygosity for p.Gly1752Arg with recessive HL in multiple affected subjects from several unrelated families; (ii) significantly higher frequency of c.5254G>A in patients compared with ethnically matched controls. Thus, we have provided sufficient genetic evidence for possible association of this variant with the disease [48,49]. However, only two out of four (PolyPhen2, LRT) mutation prediction tools used in this study defined p.Gly1752Arg as possibly damaging or deleterious. Therefore, in future studies, it would be necessary to investigate the effect of this variant on the protein structure and function, as well as to elucidate the biological role of the *RAI1* gene in the development of HL. Although the direct involvement of this gene in HL is not yet established, there are some promising indications to this association. *RAI1* (retinoic acid induced 1) is inducible by retinoic acid [50] and the retinoic acid signaling is known to mediate the complicated pathways of the mammalian organ of Corti development [51–55]. We suggest that putative involvement of the *RAI1* gene in HL could be related to this mechanism. We aim to clarify these issues and prove presumed pathogenicity of the p.Gly1752Arg variant in further experimental studies beyond the scope of this work.

Currently, interest in study of *RAI1* gene has rapidly increased due to its association not only with Smith-Magenis syndrome but also with some other neurodegenerative and neuropsychiatric disorders [28–33]. In this connection, the *RAI1* gene studies are generally restricted to examinations of specific groups of patients. We believe that presumed association of the *RAI1* mutation with isolated HL broadens the spectrum of clinical features associated with *RAI1* mutations and will draw attention for potential involvement of this poorly studied gene in complicated auditory pathways.

The *OTOF* gene, consisting of 48 exons, encodes the transmembrane protein otoferlin. Otoferlin plays an important role in vesicle release at the synapse between inner hair cells and auditory nerve fibers through a Ca^2+^-dependent interaction with surrounding proteins at the auditory ribbon synapse [56,57]. Currently, ~ 90 mutations in *OTOF* (see the most complete list in review [58]) have been reported to cause a nonsyndromic severe-to-profound prelingual HL and autosomal recessive auditory neuropathy-1 (DFNB9, deafness, autosomal recessive 9, AUNB1, MIM 601071) characterized by disruption of auditory nerve activity with preservation of outer hair cell function.

Previously unreported (absent in genome databases) (Table 2) missense mutation c.1111C>G in exon 12 of *OTOF* leads to substitution Gly-to-Arg at evolutionarily conserved amino acid position 371 (p.Gly371Arg) in the region between domains C2B and C2C of OTOF (S3 Fig). Notably, mutations c.1103_1104delinsC (p.G368AfsX2), c.1180dupG (p.E394GfsX6), c.1194T>A (p.D398E), and c.1236delC (p.E413NfsX90) located in the same region of the OTOF protein have been earlier reported in patients with severe-profound HL [59–61].

Variant c.1111C>G found in this study has been predicted ‘probably damaging’, ‘damaging’, ‘disease_causing’ or ‘deleterious’ by PolyPhen2, SIFT, Mutation Taster, and LRT, respectively (Table 2). We also found segregation of homozygosity for c.1111C>G with recessive HL in Altaian family F54: all c.1111C>G homozygous siblings from this family were affected and all tested normal hearing relatives were either wt/wt or heterozygous for c.1111C>G (Fig 2). Altogether, these data support plausible association of homozygosity for c.1111C>G with recessive HL in Altaian patients. This statement is challenged by presence of affected F24 members from the combined pedigree F24-F54 (Fig 2) with one c.1111C>G copy (24-II-2) or without c.1111C>G (24–1 and 24-II-1). Presence of other deleterious allele in compound with c.1111C>G seems unlikely in patient 24-II-2 due to a very low probability of co-occurrence of rare deleterious *OTOF* variants in extended F24-F54 family from small isolated Altaian population. Such possibility can not be excluded without costly and labor-intensive sequencing of all 48 exons of the *OTOF* gene in patient 24-II-2. Here we believe that development of HL in family F24 is more likely explained by either untested genetic or environmental factors.

Mutations in the *SLC26A4* gene are the second most frequent cause of human hereditary HL, after mutations in the *GJB2* gene, accounting for ~ 10% of all hereditary hearing impairment cases. To date, more than 160 *SLC26A4* mutations have been identified (Pendred/BOR Homepage, http://www.healthcare.uiowa.edu/labs/pendredandbor/), and the spectrum of common *SLC26A4* mutations differs across populations. Mutation c.2168A>G (p.His723Arg) found in Altaian family F53 is one of the most prevalent mutations in both recessive NSHL and PDS families in the Asian populations [62–64].

**Fig 3:**
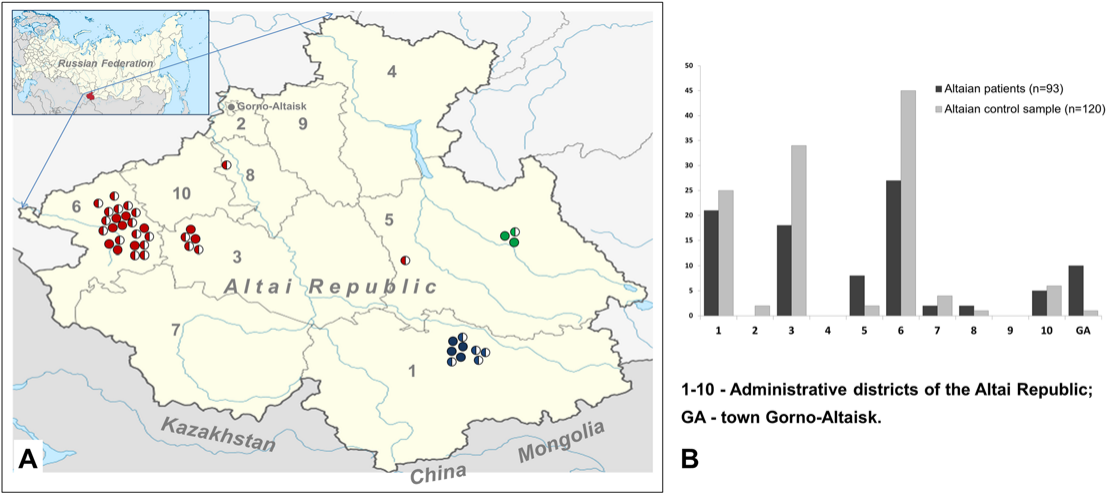
Prevalence of mutations c.5254G>A (*RAI1*), c.1111C>G (*OTOF*), and c.2168A>G (*SLC26A4*) on the territory of the Altai Republic. (**A**) Territorial distribution of individuals homozygous or heterozygous for mutations denoted by fully or half-colored circle, correspondingly: c.5254G>A (*RAI1*)–by red; c.1111C>G (*OTOF*)–by blue; c.2168A>G (*SLC26A4*)–by green. (**B**) Distribution of studied Altaian patients (n = 93) and Altaian control sample (n = 120) on the territory of the Altai Republic.

Distribution of all alleles with mutations c.5254G>A (*RAI1*), c.1111C>G (*OTOF*), and c.2168A>G (*SLC26A4*) identified in Altaian patients, their tested relatives, and heterozygous carriers on the Altai Republic territory is presented in Fig 3. The Altai Republic, bordering Mongolia, China, and Kazakhstan is inhabited by ~ 200,000 people including Altaians, Russians, Kazakhs and other ethnicities. The Altaians (~ 60,000), indigenous inhabitants of the Altai region, originate from several ancient Turkic-speaking tribes [65]. Contemporary territory of the Altai Republic is subdivided into ten administrative rural districts with the boundaries approximately corresponding to ancestral Altaian clans’ lands. Accumulation of c.5254G>A (*RAI1*) in two neighboring north-western districts probably reflects the founder effect in the particular Altaian clan subgroups who traditionally occupied these territories. Prevalence of c.1111C>G (*OTOF*) is restricted to one extended Altaian family F54-F24 living in south-eastern district of the Altai Republic, and mutation c.2168A>G (*SLC26A4*) was found in the only Altaian family F53 residing in eastern region of the Altai Republic. The geographically specific distribution of these discovered variants may imply a founder effect in the indigenous populations of the Altai region.

## Conclusions

Our data confirm the utility of whole exome sequencing (WES) for successful identification of candidate causal variants in some Altaian families with autosomal recessive NSHL of unknown genetic etiology. Through WES, we identified missense mutations in three genes (*RAI1*, *OTOF*, and *SLC26A4*) associated with HL. Several lines of evidences support the association of homozygosity for identified variants c.5254G>A (*RAI1*), c.1111C>G (*OTOF*), and c.2168A>G (*SLC26A4*) with HL in Altaian patients. Local prevalence of uncovered variants implies possible founder effect in sufficient number of HL cases in indigenous population of the Altai region. Notably, this study is the first report about patients with *RAI1* mutations whose HL is not accompanied by specific traits typical for Smith-Magenis syndrome. Presumed association of *RAI1* gene variant c.5254G>A with isolated HL needs to be proved by further experimental studies.

## Supporting Information

S1 Fig Evolutionary conservation (on nucleotide and amino acid level) for the positions of detected variants. (PDF)

S2 Fig Schematic representation of the *RAI1* gene with 6 exons and summarized point mutations found in SMS patients without deletion in 17p11.2. (PDF)

S3 Fig Schematic structure of the otoferlin (OTOF) protein with reported mutations. (PDF)

S1 Table Patients with HL from four Altaian families (F38, F40, F53, F54) analyzed by WES. (PDF)

S2 Table Primers for PCR / Sanger sequencing. (PDF)

S3 Table The *RAI1* genotypes with allelic variations detected by Sanger sequencing in individuals homozygous and heterozygous for c.5254G>A. (PDF)

S4 Table Mutations identified in the *RAI1* gene in the SMS patients without any 17p11.2 deletions (literature data) and in patients with isolated HL (this study). (PDF)
