# Supplementary material for: Whole Exome Sequencing Reveals Homozygous Mutations in RAI1, OTOF, and SLC26A4 Genes Associated with Nonsyndromic Hearing Loss in Altaian Families (South Siberia)
Source: PLoS One. 2016 Apr 15;11(4):e0153841. doi: 10.1371/journal.pone.0153841 (PMC4833413; doi:10.1371/journal.pone.0153841)
Supplement: S1 Fig — (PDF) [file pone.0153841.s001.pdf]

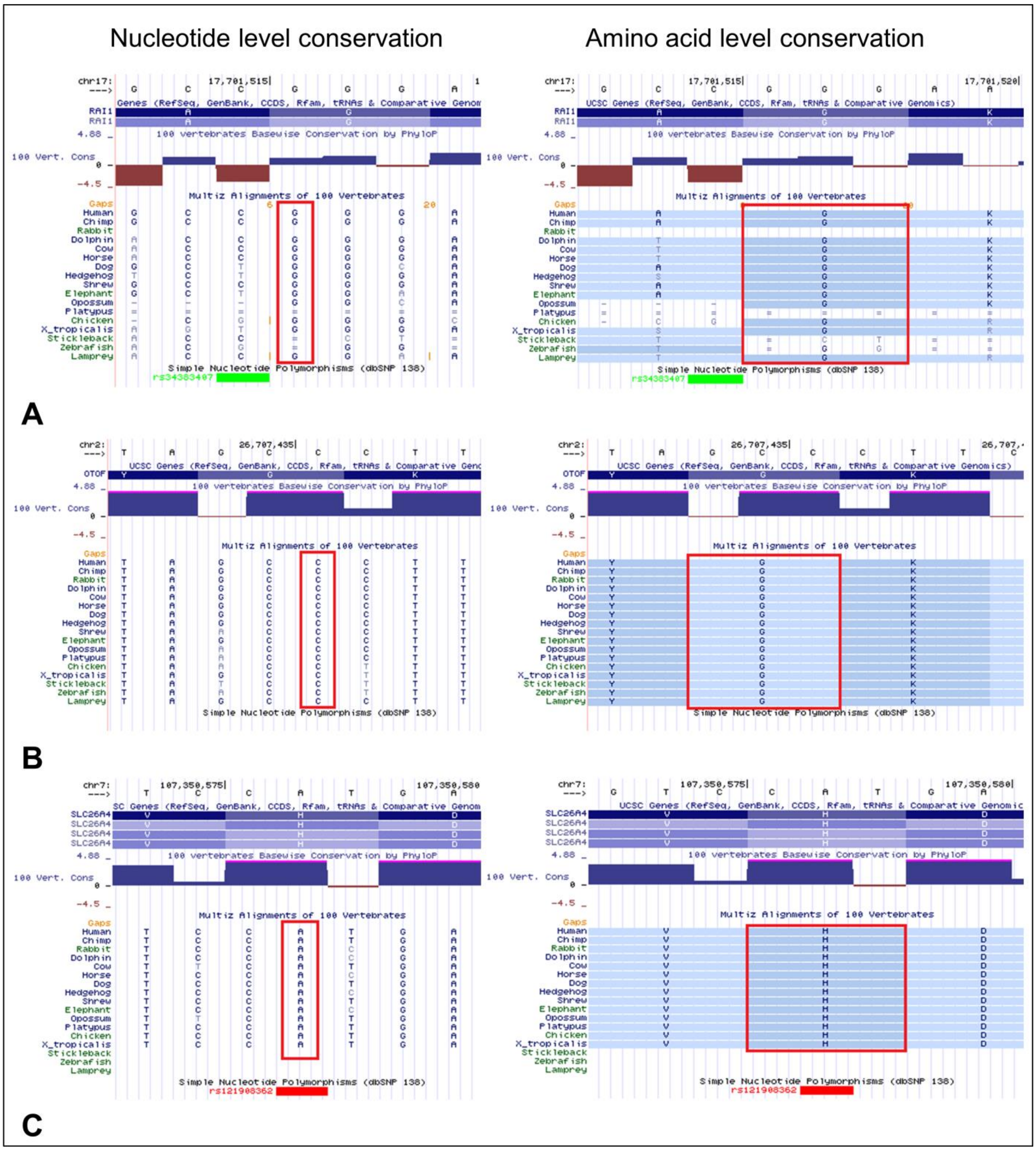

S1 Fig. Evolutionary conservation (on nucleotide and amino acid level) for the positions of detected variants.

(**A**) *RAI1* (retinoic acid induced gene 1), chr17:17701516: c.5254G>A (p.Gly1752Arg). Other amino acids (but never arginine) can present in corresponding position in some organisms: Zebra Finch, Tibetan Ground Jay, Rock pigeon, American Allegator, Lizard, Big brown bat, Microbat, David's myotis and some others (data not shown).

(**B**) *OTOF* (otoferlin), chr2: 26707436: c.1111C>G (p.Gly371Arg). (**C**) *SLC26A4* (pendrin), chr7: 107350577: c.2168A>G (p.His723Arg).

(100 vertebrate multiple alignment hosted by UCSC genome browser: https://genome.ucsc.edu).
