# Supplementary material for: Whole Exome Sequencing Reveals Homozygous Mutations in RAI1, OTOF, and SLC26A4 Genes Associated with Nonsyndromic Hearing Loss in Altaian Families (South Siberia)
Source: PLoS One. 2016 Apr 15;11(4):e0153841. doi: 10.1371/journal.pone.0153841 (PMC4833413; doi:10.1371/journal.pone.0153841)
Supplement: S2 Fig — (PDF) [file pone.0153841.s002.pdf]

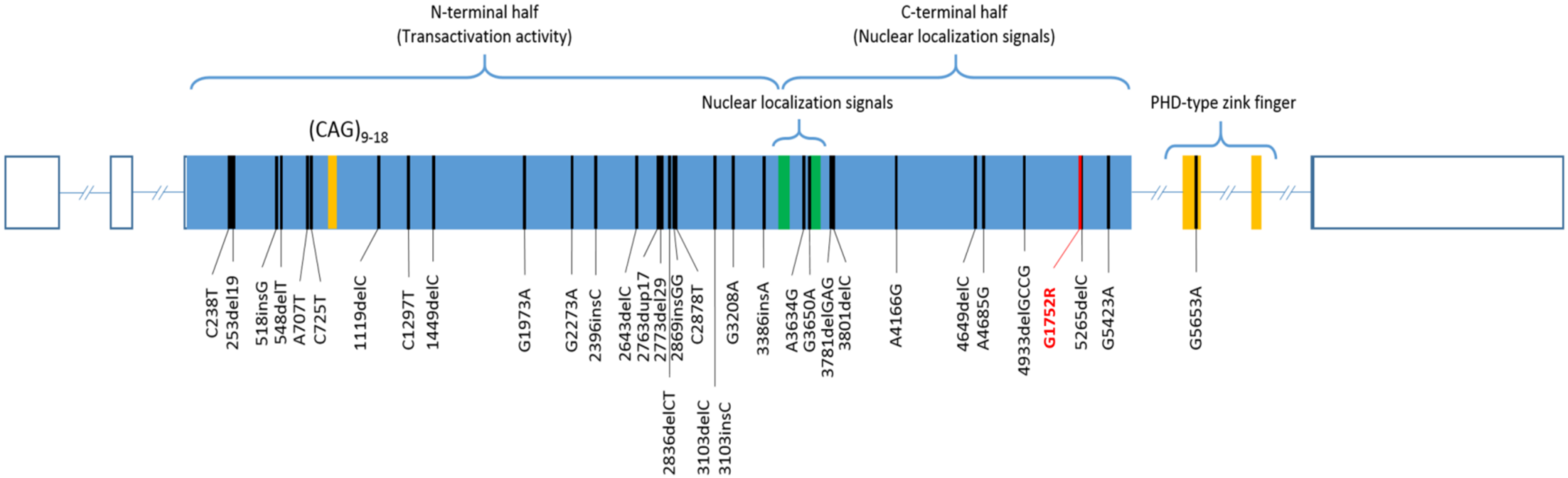

S2 Fig. Schematic representation of the *RAI1* gene with 6 exons and summarized point mutations found in SMS patients without deletion in 17p11.2.

Data on point *RAI1* mutations have been taken from appropriate papers [1-10]. The majority of reported *RAI1* mutations are located in exon 3 (blue box) containing ~ 98% of all *RAI1* gene coding sequence. Mutation c.5254G>A (p.Gly1752Arg [p.G1752R]) identified in present study is shown in red. $(CAG)_{9\text{-}18}$ – the range of CAG repeats earlier detected in SMS patients. Variability in the polymorphic CAG repeat length in the range of 10-16 repeats is common for general population [8] and apparently does not affect the SMS phenotype [1]. However, in study Bi *et al* (2006) was shown that combination of large $(CAG)_{18}$ repeat with c.3634A>G mutation in one patient could increase expression of the SMS phenotype [8].

References for S2 Fig

1. Dubourg C, Bonnet-Brilhault F, Toutain A, Mignot C, Jacquette A, Dieux A, et al. Identification of Nine New RAI1-Truncating Mutations in Smith-Magenis Syndrome Patients without 17p11.2 Deletions. Mol Syndromol. 2014; 5(2): 57-64. doi: 10.1159/000357359

2. Girirajan S, Elsas LJ 2nd, Devriendt K, Elsea SH. RAI1 variations in Smith-Magenis syndrome patients without 17p11.2 deletions. J Med Genet. 2005;42(11): 820-828. doi: 10.1136/jmg.2005.031211

3. Vilboux T, Ciccone C, Blancato JK, Cox GF, Deshpande C, Introne WJ, et al. Molecular analysis of the Retinoic Acid Induced 1 gene (RAI1) in patients with suspected Smith-Magenis syndrome without the 17p11.2 deletion. PLoS One. 2011; 6(8): e22861. doi: 10.1371/journal.pone.0022861

4. Girirajan S, Vlangos CN, Szomju BB, Edelman E, Trevors CD, Dupuis L, et al. Genotype-phenotype correlation in Smith-Magenis syndrome: evidence that multiple genes in 17p11.2 contribute to the clinical spectrum. Genet Med. 2006; 8(7): 417-427.

5. Slager RE, Newton TL, Vlangos CN, Finucane B, Elsea SH. Mutations in RAI1 associated with Smith-Magenis syndrome. Nat Genet. 2003; 33(4): 466-468. doi: 10.1038/ng1126

6. Adams DR, Yuan H, Holyoak T, Arajs KH, Hakimi P, Markello TC, et al. Three rare diseases in one Sib pair: RAI1, PCK1, GRIN2B mutations associated with Smith-Magenis Syndrome, cytosolic PEPCK deficiency and NMDA receptor glutamate insensitivity. Mol Genet Metab. 2014; 113(3): 161-170. doi: 10.1016/j.ymgme.2014.04.001

7. Bi W, Saifi GM, Shaw CJ, Walz K, Fonseca P, Wilson M, et al. Mutations of RAI1, a PHD-containing protein, in nondeletion patients with Smith-Magenis syndrome. Hum Genet. 2004; 115(6): 515-524.

8. Bi W, Saifi GM, Girirajan S, Shi X, Szomju B, Firth H, et al. RAI1 point mutations, CAG repeat variation, and SNP analysis in non-deletion Smith-Magenis syndrome. Am J Med Genet A. 2006; 140(22): 2454-2463. doi: 10.1002/ajmg.a.31510

9. Truong HT, Dudding T, Blanchard CL, Elsea SH. Frameshift mutation hotspot identified in Smith-Magenis syndrome: case report and review of literature. BMC Med Genet. 2010; 11: 142. doi: 10.1186/1471-2350-11-142

10. Vieira GH, Rodriguez JD, Carmona-Mora P, Cao L, Gamba BF, Carvalho DR, et al. Detection of classical 17p11.2 deletions, an atypical deletion and RAI1 alterations in patients with features suggestive of Smith-Magenis syndrome. Eur J Hum Genet. 2012; 20(2): 148-154. doi: 10.1038/ejhg.2011.167
