# Supplementary material for: Whole Exome Sequencing Reveals Homozygous Mutations in RAI1, OTOF, and SLC26A4 Genes Associated with Nonsyndromic Hearing Loss in Altaian Families (South Siberia)
Source: PLoS One. 2016 Apr 15;11(4):e0153841. doi: 10.1371/journal.pone.0153841 (PMC4833413; doi:10.1371/journal.pone.0153841)
Supplement: S3 Fig — (PDF) [file pone.0153841.s003.pdf]

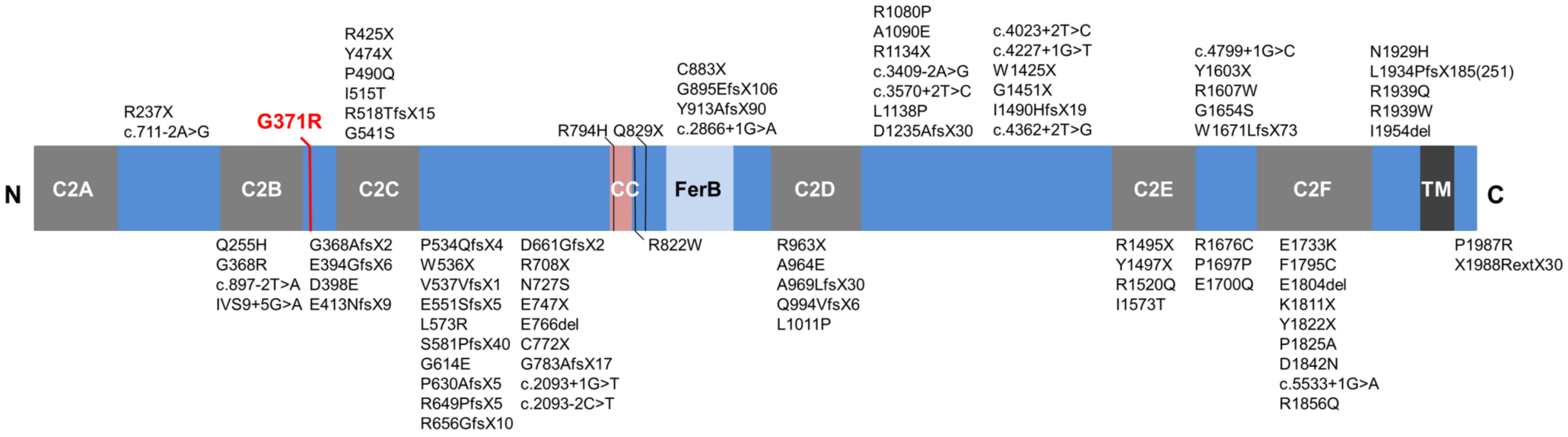

S3 Fig. Schematic structure of the otoferlin (OTOF) protein with reported mutations.

N - (NH2) terminus of the OTOF protein; C - carboxyl (COOH) terminus; TM - transmembrane domain (aa 1964–1984); FerB - Ferlin-specific motif; C2 domains: C2A (aa 1–97), C2B (aa 254–352), C2C (aa 417–528); C2D (aa 960–1067), C2E (aa 1493–1592), and C2F (aa 1733–1863); CC - coiled coil domain (aa 792–821). Inter domains regions are shown in blue. The mutation p.G371R (p.Gly371Arg [c.1111C>G]) identified in present study is shown in red.
