# Supplementary material for: Whole Exome Sequencing Reveals Homozygous Mutations in RAI1, OTOF, and SLC26A4 Genes Associated with Nonsyndromic Hearing Loss in Altaian Families (South Siberia)
Source: PLoS One. 2016 Apr 15;11(4):e0153841. doi: 10.1371/journal.pone.0153841 (PMC4833413; doi:10.1371/journal.pone.0153841)
Supplement: S1 Table — (PDF) [file pone.0153841.s004.pdf]

S1 Table. Patients with HL from four Altaian families (F38, F40, F53, F54) analyzed by WES.

Family and patient code	Hearing status	Year of birth	Sex	Ethnicity
**F38:**				
38-II-4	congenital profound sensorineural HL	1964	female	Altaian
38-II-5	congenital profound sensorineural HL	1966	male	Altaian
**F40:**				
40-II-1	profound mixed (sensorineural and conductive) HL	1960	female	Altaian
40-II-3	congenital profound sensorineural HL	1979	female	Altaian
**F53:**				
53-II-1	congenital profound sensorineural HL	1988	male	Altaian
**F54:**				
54-II-2	congenital profound sensorineural HL	1975	female	Altaian
54-II-5	congenital profound sensorineural HL	1982	female	Altaian

HL- hearing loss; WES – whole exome sequencing.
