# Supplementary material for: Whole Exome Sequencing Reveals Homozygous Mutations in RAI1, OTOF, and SLC26A4 Genes Associated with Nonsyndromic Hearing Loss in Altaian Families (South Siberia)
Source: PLoS One. 2016 Apr 15;11(4):e0153841. doi: 10.1371/journal.pone.0153841 (PMC4833413; doi:10.1371/journal.pone.0153841)
Supplement: S2 Table — (PDF) [file pone.0153841.s005.pdf]

S2 Table. Primers for PCR / Sanger sequencing.

Mutation	Primers	Product size
c.5254G>A (*RAI1*)	RAI-U: 5'-TCCTTGCCCCTCTCCTCCAC-3'<br>RAI-L: 5'-GCCTCCTTGCTGCACTCATGTTT-3'	446
c.1111C>G (*OTOF)*	OTOF-U: 5'-CGGAAGAGTGGGGCTGCTTG-3'<br>OTOF-L: 5'-CTCTAGGGACCAAGACAGCATTT-3'	417
c.2168A>G (*SLC26A4*)	Ex19_F: 5'-CGTGATCGTCCACAAG-3'<br>Ex19_R: 5'-GGGTACTACCAGGTAATTT-3'	754
