# Supplementary material for: Whole Exome Sequencing Reveals Homozygous Mutations in RAI1, OTOF, and SLC26A4 Genes Associated with Nonsyndromic Hearing Loss in Altaian Families (South Siberia)
Source: PLoS One. 2016 Apr 15;11(4):e0153841. doi: 10.1371/journal.pone.0153841 (PMC4833413; doi:10.1371/journal.pone.0153841)
Supplement: S3 Table — (PDF) [file pone.0153841.s006.pdf]

S3 Table. The *RAI1* genotypes with allelic variations detected by Sanger sequencing in individuals homozygous and heterozygous for c.5254G>A.

Code	Hearing status	Genotypes					Allele
		**rs3803763** c.269G>C p.Gly90Ala	**rs11649804** c.493C>A p.Pro165Thr	**Variations in polyQ region ***	**rs8067439** c.1992G>A p.Pro664Pro	**Mutation** c.5254G>A p.Gly1752Arg	
**Homozygotes for c.5254G>A**							
**38-II-5**	HL	C/C	A/A	Q_{13}[CAG CAA (CAG)$_{10}$ del(CAG) CAA] / Q_{13}[CAG CAA (CAG)$_{10}$ del(CAG) CAA]	G/G	A/A	Allele$_{mut}$: C-A-Q_{13}[CAG CAA (CAG)$_{10}$ del(CAG) CAA]-G-A<br>Allele$_{mut}$: C-A-Q_{13}[CAG CAA (CAG)$_{10}$ del(CAG) CAA]-G-A
**40-II-1**	HL	C/C	A/A	Q_{13}[CAG CAA (CAG)$_{10}$ del(CAG) CAA] / Q_{13}[CAG CAA (CAG)$_{10}$ del(CAG) CAA]	G/G	A/A	Allele$_{mut}$: C-A-Q_{13}[CAG CAA (CAG)$_{10}$ del(CAG) CAA]-G-A<br>Allele$_{mut}$: C-A-Q_{13}[CAG CAA (CAG)$_{10}$ del(CAG) CAA]-G-A
**42-II-1**	HL	C/C	A/A	Q_{13}[CAG CAA (CAG)$_{10}$ del(CAG) CAA] / Q_{13}[CAG CAA (CAG)$_{10}$ del(CAG) CAA]	G/G	A/A	Allele$_{mut}$: C-A-Q_{13}[CAG CAA (CAG)$_{10}$ del(CAG) CAA]-G-A<br>Allele$_{mut}$: C-A-Q_{13}[CAG CAA (CAG)$_{10}$ del(CAG) CAA]-G-A
**43-III-1**	HL	C/C	A/A	Q_{13}[CAG CAA (CAG)$_{10}$ del(CAG) CAA] / Q_{13}[CAG CAA (CAG)$_{10}$ del(CAG) CAA]	G/G	A/A	Allele$_{mut}$: C-A-Q_{13}[CAG CAA (CAG)$_{10}$ del(CAG) CAA]-G-A<br>Allele$_{mut}$: C-A-Q_{13}[CAG CAA (CAG)$_{10}$ del(CAG) CAA]-G-A
**Affected heterozygotes for c.5254G>A**							
**18-II-1**	HL	G/C	C/A	Q_{13} [CAG CAA (CAG)$_{10}$ del(CAG) CAA] / Q_{14} [CAG CAG (CAG)$_{11}$ CAA]	G/A	G/A	Allele$_{mut}$: C-A-Q_{13}[CAG CAA (CAG)$_{10}$ del(CAG) CAA]-G-A<br>Allele$_{1}$: G-C-Q_{14}[CAG CAG (CAG)$_{11}$ CAA]-A-G
**37-II-1**	HL	G/C	C/A	Q_{13}[CAG CAA (CAG)$_{10}$ del(CAG) CAA] / Q_{13}[CAG CAG (CAG)$_{10}$ del(CAG) CAA]	G/A	G/A	Allele$_{mut}$: C-A-Q_{13}[CAG CAA (CAG)$_{10}$ del(CAG) CAA]-G-A<br>Allele$_{2}$: G-C-Q_{13}[CAG CAG (CAG)$_{10}$ del(CAG) CAA]-A-G
**38-I-1**	HL	C/C	A/A	Q_{13}[CAG CAA (CAG)$_{10}$ del(CAG) CAA] / Q_{13}[CAG CAA (CAG)$_{10}$ del(CAG) CAA]	G/G	G/A	Allele$_{mut}$: C-A-Q_{13}[CAG CAA (CAG)$_{10}$ del(CAG) CAA]-G-A<br>Allele$_{3}$: C-A-Q_{13}[CAG CAA (CAG)$_{10}$ del(CAG) CAA]-G-G
**43-II-2**	HL	C/C	A/A	Q_{13}[CAG CAA (CAG)$_{10}$ del(CAG) CAA / Q_{13}[CAG CAA (CAG)$_{10}$ del(CAG) CAA]	G/G	G/A	Allele$_{mut}$: C-A-Q_{13}[CAG CAA (CAG)$_{10}$ del(CAG) CAA]-G-A<br>Allele$_{3}$: C-A-Q_{13}[CAG CAA (CAG)$_{10}$ del(CAG) CAA]-G-G
**Normal hearing heterozygotes for c.5254G>A**							
**37-I-2**	normal hearing	C/C	A/A	Q_{13}[CAG CAA (CAG)$_{10}$ del(CAG) CAA] / Q_{13}[CAG CAA (CAG)$_{10}$ del(CAG) CAA]	G/G	G/A	Allele$_{mut}$: C-A-Q_{13}[CAG CAA (CAG)$_{10}$ del(CAG) CAA]-G-A<br>Allele$_{3}$: C-A-Q_{13}[CAG CAA (CAG)$_{10}$ del(CAG) CAA]-G-G
**43-II-3**	normal hearing	C/C	A/A	Q_{13}[CAG CAA (CAG)$_{10}$ del(CAG) CAA] / Q_{13}[CAG CAA (CAG)$_{10}$ del(CAG) CAA]	G/G	G/A	Allele$_{mut}$: C-A-Q_{13}[CAG CAA (CAG)$_{10}$ del(CAG) CAA]-G-A<br>Allele$_{3}$: C-A-Q_{13}[CAG CAA (CAG)$_{10}$ del(CAG) CAA]-G-G
**38-II-1**	normal hearing	C/C	A/A	Q_{13}[CAG CAA (CAG)$_{10}$ del(CAG) CAA] / Q_{13}[CAG CAA (CAG)$_{10}$ del(CAG) CAA]	G/G	G/A	Allele$_{mut}$: C-A-Q_{13}[CAG CAA (CAG)$_{10}$ del(CAG) CAA]-G-A<br>Allele$_{3}$: C-A-Q_{13}[CAG CAA (CAG)$_{10}$ del(CAG) CAA]-G-G
**Alt-1**	normal hearing	C/C	A/A	Q_{13}[CAG CAA (CAG)$_{10}$ del(CAG) CAA] / Q_{13}[CAG CAA (CAG)$_{10}$ del(CAG) CAA]	G/G	G/A	Allele$_{mut}$: C-A-Q_{13}[CAG CAA (CAG)$_{10}$ del(CAG) CAA]-G-A<br>Allele$_{3}$: C-A-Q_{13}[CAG CAA (CAG)$_{10}$ del(CAG) CAA]-G-G
**Alt-2**	normal hearing	C/C	C/A	Q_{13}[CAG CAA (CAG)$_{10}$ del(CAG) CAA] / Q_{11}[CAG CAG (CAG)$_{8}$ del(CAG)$_{3}$ CAA]	G/A	G/A	Allele$_{mut}$: C-A-Q_{13}[CAG CAA (CAG)$_{10}$ del(CAG) CAA]-G-A<br>Allele$_{4}$: C-C- Q_{11}[CAG CAG (CAG)$_{8}$ del(CAG)$_{3}$ CAA]-A-G

HL – hearing loss, * NM_030665 ref seq.: (CAG)$_{13}$ CAA=Q_{14}; variations in polyQ region (starting from 832 nucleotide position) includes rs11078398: c.837G>A (caG/caA) p.Gln279Gln; codon deletions: del(CAG) and del(CAG)$_{3}$. The common haplotype for c.5254G>A (allele$_{mut}$) is highlighted by yellow, allele$_{1}$, allele$_{2}$, allele$_{3}$, and allele$_{4}$ - by grey, pink, blue, and green, respectively. Nucleotides embedded in common haplotype for c.5254G>A (allele$_{mut}$) are shown by red.
