# Supplementary material for: Whole Exome Sequencing Reveals Homozygous Mutations in RAI1, OTOF, and SLC26A4 Genes Associated with Nonsyndromic Hearing Loss in Altaian Families (South Siberia)
Source: PLoS One. 2016 Apr 15;11(4):e0153841. doi: 10.1371/journal.pone.0153841 (PMC4833413; doi:10.1371/journal.pone.0153841)
Supplement: S4 Table — (PDF) [file pone.0153841.s007.pdf]

S4 Table. Mutations identified in the *RAI1* gene in the SMS patients without any 17p11.2 deletions (literature data) and in patients with isolated HL (this study).

Nucleotide change	Mutation type	Amino acid change	(CAG)-repeats at 832 nucleotide position	Familial or *de novo*	Number (code) of patients	Common SMS clinical symptoms / Hearing status	References
			**N-terminal part of the RAI1 protein**				
c.238C>T	nonsense	p.Arg80*	$(CAG)_{13-14}$	no data	1 (RNS78)	+ / normal hearing	[1]
c.253del19	frameshift	p.Leu85fsX60	$(CAG)_{13}/(CAG)_{13}$	*de novo*	1 (SMS153)	+ / normal hearing	[2]
c.518_519insG	frameshift	p.Gln174Profs*64	$(CAG)_{13-14}$	no data	1 (RNS103)	+ / normal hearing	[1]
c.548delT	frameshift	p.L183RfsX69	$(CAG)_{14}/(CAG)_{14}$	*de novo*	1 (M2911)	+ / HL	[3]
c.707A>T	missense	p.Y236F	$(CAG)_{14}/(CAG)_{14}$	familial	1 (M2732)	+ / normal hearing	[3]
c.725C>T and c.2907C>T [a]	missense	p.P242L and p.D969D	$(CAG)_{13}/(CAG)_{13}$	unclassified	1 (M2543)	+ / no data	[3]
c.1119delC	frameshift	p.Ser373fsX65	NA	*de novo*	1 (SMS201)	+ / normal hearing	[4]
c.1297C>T	nonsense	p.Gln433*	$(CAG)_{13-14}$	no data	1 (RNS105)	+ / normal hearing	[1]
c.1449delC (c.1308delC)	frameshift	p.E484KfsX35	$(CAG)_{14}/(CAG)_{14}$	*de novo*	1 (M2377=SMS159)	+ / HL	[3,5]
c.1500G>A [a] and c.3791A>G (rs61746214)	missense	p.P500P and p.E1264G	$(CAG)_{14}/(CAG)_{14}$	familial	1 (M2900)	+ / normal hearing	[3]
c.1973G>A	nonsense	W658X	$(CAG)_{13}/(CAG)_{13}$	*de novo*	1 (M2719)	+ / HL	[3]
c.2273G>A	nonsense	p.W758X	NA	*de novo*	1 (patient 1)	+ / no data	[6]
c.2396dupC	frameshift	p.Gly800Trpfs*36	$(CAG)_{13-14}$	no data	1 (RNS165)	+ / normal hearing	[1]
c.2643delC	frameshift	p.Glu882Serfs*68	$(CAG)_{13-14}$	no data	1 (RNS86)	+ / normal hearing	[1]
c.2763_2779dup17	frameshift	p.Leu927Glnfs*29	$(CAG)_{13-14}$	no data	1 (RNS131)	+ / normal hearing	[1]
c.2773del29	frameshift	p.Val925fsX8	$(CAG)_{13}/(CAG)_{13}$	*de novo*	1 (SMS129=BAB539)	+ / normal hearing	[5,7]
c.2836_2837delCT	frameshift	p.Leu946Valfs*7	$(CAG)_{13-14}$	no data	1 (RNS208)	+ / normal hearing	[1]
c.2869_2870insGG	frameshift	p.Asp957Glyfs*108	$(CAG)_{13-14}$	no data	1 (RNS101)	+ / normal hearing	[1]
c.2878C>T	nonsense	p.Arg960X	$(CAG)_{10}/(CAG)_{11}$	no data	1 (BAB1106)	+ / normal hearing	[7]
c.3103delC	frameshift	p.Gln1035fsX28	$(CAG)_{10}/(CAG)_{10}$<br>NA	*de novo*<br>unclassified	1 (BAB2416)<br>1 (SMS324)	+ / normal hearing<br>+ / HL	[8]<br>[9]
c.3103insC	frameshift	p.Q1034PfsX31 (p.Gln1035fsX30)	$(CAG)_{10}/(CAG)_{11}$<br>NA	*de novo*<br>unclassified	1 (M2754=BAB1852)<br>1 (SMS335)	+ / HL<br>+ / HL	[3,7]<br>[9]

S4 Table. Mutations identified in the *RAI1* gene in the SMS patients without any 17p11.2 deletions (literature data) and in patients with isolated HL (this study). (continued)

Nucleotide change	Mutation type	Amino acid change	(CAG)-repeats at 832 nucleotide position	Familial or *de novo*	Number (code) of patients	Common SMS clinical symptoms / Hearing status	References
			**C-terminal part of the RAI1 protein**				
c.3183G>A [a] and c.5653G>A [b]	missense	p.T1061T and p.D1885N	$(CAG)_{13}/(CAG)_{13}$	familial	1 (M2365)	+ / HL	[3]
c.3208G>A and c.4512G>T [a]	missense	p.G1070R and p.L1504L	$(CAG)_{14}/(CAG)_{14}$	familial	1 (M2826)	+ / HL	[3]
c.3386dupA	frameshift	p.Glu1130Glyfs*36	$(CAG)_{13-14}$	*de novo*	1 (RNS59)	+ / normal hearing	[1]
c.3634A>G	missense	p.Ser1212Gly	$(CAG)_{13}/(CAG)_{18}$	familial	1 (BAB2330)	+ / normal hearing	[8]
c.3650G>A	missense	R1217Q	NA	unclassified	1 (SAG4739)	+ / no data	[10]
c.3781_3783delGAG	frameshift	p.del1261E	$(CAG)_{14}/(CAG)_{14}$	familial	1 (M2867)	+ / normal hearing	[3]
c.3801delC	frameshift	p.Pro1267fsX46	$(CAG)_{10}/(CAG)_{11}$	*de novo*	1 (SMS188)	+ / normal hearing	[2]
c.4166A>G	missense	Q1389R	NA	unclassified	1 (SAG6888)	+ / no data	[10]
c.4649delC	frameshift	p.Ser1550fsX36	NA	*de novo*	1 (SMS278)	+ / normal hearing	[4]
c.4685A>G	missense	p.Gln1562Arg	$(CAG)_{9}/(CAG)_{11}$	*de novo*	1 (SMS175)	+ / normal hearing	[2]
c.4933delGCCG	frameshift	p.Ala1645fsX35	NA	*de novo*	1 (SMS300)	+ / normal hearing	[4]
**c.5254G>A**	missense	**p.Gly1752Arg**	$(CAG)_{13}/(CAG)_{13}$	familial	14 (10 homozygotes and 4 heterozygotes)	- / HL	**this study**
c.5265delC (c.4929delC)	frameshift	p.Pro1755fsX74	$(CAG)_{10}/(CAG)_{11}$	*de novo*	1 (SMS156=BAB526)	+ / HL	[5,7]
c.5423G>A	missense	p.Ser1808Asn	$(CAG)_{10}/(CAG)_{11}$	unclassified	1 (SMS195)	+ / normal hearing	[2]

HL – hearing loss. [a] - these silent variants are probably not related to the phenotype SMS [3]; [b] - variant in exon 4; NA – not analyzed

References for S4 Table

1. Dubourg C, Bonnet-Brilhault F, Toutain A, Mignot C, Jacquette A, Dieux A, et al. Identification of Nine New RAI1-Truncating Mutations in Smith-Magenis Syndrome Patients without 17p11.2 Deletions. Mol Syndromol. 2014; 5(2): 57-64. doi: 10.1159/000357359

2. Girirajan S, Elsas LJ 2nd, Devriendt K, Elsea SH. RAI1 variations in Smith-Magenis syndrome patients without 17p11.2 deletions. J Med Genet. 2005;42(11): 820-828. doi: 10.1136/jmg.2005.031211

3. Vilboux T, Ciccone C, Blancato JK, Cox GF, Deshpande C, Introne WJ, et al. Molecular analysis of the Retinoic Acid Induced 1 gene (RAI1) in patients with suspected Smith-Magenis syndrome without the 17p11.2 deletion. PLoS One. 2011; 6(8): e22861. doi: 10.1371/journal.pone.0022861

4. Girirajan S, Vlangos CN, Szomju BB, Edelman E, Trevors CD, Dupuis L, et al. Genotype-phenotype correlation in Smith-Magenis syndrome: evidence that multiple genes in 17p11.2 contribute to the clinical spectrum. Genet Med. 2006; 8(7): 417-427.

5. Slager RE, Newton TL, Vlangos CN, Finucane B, Elsea SH. Mutations in RAI1 associated with Smith-Magenis syndrome. Nat Genet. 2003; 33(4): 466-468. doi: 10.1038/ng1126

6. Adams DR, Yuan H, Holyoak T, Arajs KH, Hakimi P, Markello TC, et al. Three rare diseases in one Sib pair: RAI1, PCK1, GRIN2B mutations associated with Smith-Magenis Syndrome, cytosolic PEPCK deficiency and NMDA receptor glutamate insensitivity. Mol Genet Metab. 2014; 113(3): 161-170. doi: 10.1016/j.ymgme.2014.04.001

7. Bi W, Saifi GM, Shaw CJ, Walz K, Fonseca P, Wilson M, et al. Mutations of RAI1, a PHD-containing protein, in nondeletion patients with Smith-Magenis syndrome. Hum Genet. 2004; 115(6): 515-524.

8. Bi W, Saifi GM, Girirajan S, Shi X, Szomju B, Firth H, et al. RAI1 point mutations, CAG repeat variation, and SNP analysis in non-deletion Smith-Magenis syndrome. Am J Med Genet A. 2006; 140(22): 2454-2463. doi: 10.1002/ajmg.a.31510

9. Truong HT, Dudding T, Blanchard CL, Elsea SH. Frameshift mutation hotspot identified in Smith-Magenis syndrome: case report and review of literature. BMC Med Genet. 2010; 11: 142. doi: 10.1186/1471-2350-11-142

10. Vieira GH, Rodriguez JD, Carmona-Mora P, Cao L, Gamba BF, Carvalho DR, et al. Detection of classical 17p11.2 deletions, an atypical deletion and RAI1 alterations in patients with features suggestive of Smith-Magenis syndrome. Eur J Hum Genet. 2012; 20(2): 148-154. doi: 10.1038/ejhg.2011.167
